# The predictive value of stereotactic surgery guided by CTA angiographic point sign for secondary hematoma expansion following surgery in patients with moderate-volume basal ganglia hematoma

**DOI:** 10.3389/fneur.2025.1522598

**Published:** 2025-04-25

**Authors:** Changpin Liao, Zepeng Ni, Zhen Lu, Jiancheng Liang, Shengde Nong, Jing Ye, Xianfu Wei

**Affiliations:** ^1^Department of Neurosurgery, Baise People’s Hospital, Guangxi, China; ^2^Department of Neurosurgery, Maoming People’s Hospital, Guangdong, China; ^3^Department of Oncology Radiotherapy, Affiliated Hospital of Youjiang Medical University for Nationalities, Guangxi, China; ^4^School of Public Health, Youjiang Medical University of Nationalities, Guangxi, China

**Keywords:** point sign, moderate volume, basal ganglia hematoma, stereotactic surgery, prognosis

## Abstract

**Objective:**

To examine the efficacy of the CTA angiographic point sign in forecasting secondary hematoma expansion following stereotactic surgery in patients with moderate-volume basilar ganglia hematoma and it’s potential to enhance postoperative outcomes.

**Methods:**

A retrospective analysis was conducted on the clinical data of 143 patients with moderate-volume basal ganglia hematoma (hematoma volume between 30 mL and 60 mL) admitted to the Department of Neurosurgery at Baise People’s Hospital from January 2021 to December 2022. Stereotactic surgery guided by the CTA angiographic point sign was conducted in 79 patients (experimental group), while stereotactic surgery guided by the computed tomography (CT) scan was performed in 64 patients (control group). The short-term clinical results (incidence of secondary hematoma expansion, Glasgow Coma Scale (GCS) score within 30 days, death, surgical complications) and long-term clinical outcomes [Modified Rankin Scale (MRS) score after 6 months] were analysed by comparing the two groups.

**Results:**

No subsequent hematoma expansion occurrences transpired in the experimental group post-surgery, but 12 (18.75%) such events were observed in the control group following the procedure. The experimental group experienced 27 postoperative lung infections (34.18%), whereas the control group had 33 infections (51.56%). The average GCS score was (9.46 ± 2.23) in the experimental group and (7.94 ± 4.68) in the control group. The mortality rate was 2 (2.53%) in the experimental group and 8 (12.50%) in the control group. The treatment efficacy rate (MRS) at 6 months was 59 cases (74.68%) in the experimental group and 35 cases (54.69%) in the control group. The disparity between the two groups was statistically significant (*p* < 0.05).

**Conclusion:**

CTA angiographic point-guided stereotactic surgery can significantly diminish the incidence of subsequent hematoma expansion following the procedure, enhancing patient clinical outcomes and postoperative quality of life.

## 1 Introduction

Basal ganglia hematoma is a form of hemorrhagic stroke characterized by the rupture of small cerebral arteries, resulting in blood accumulation within the basal ganglia region. This is the predominant form of hypertensive intracerebral hemorrhage, representing roughly 50.0% of all cerebral hemorrhages, characterized by exceedingly high mortality and disability rates (1, 2). Research indicates that stereotactic surgery for cerebral hemorrhage is associated with reduced trauma, fewer postoperative complications, and expedited recovery. It offers substantial benefits in rehabilitating neurological function and enhancing clinical outcomes, making it one of the most promising surgical techniques for addressing basal ganglia hematoma (3). Currently, hematoma expansion is one of the most important factors affecting the prognosis of patients. Hematoma expansion is often categorized into primary and secondary types. The former results from active hemorrhage within the hematoma, whereas the latter arises from surgical trauma to blood vessels. Secondary hematoma expansion is a significant complication following stereotactic surgery for cerebral hemorrhage, undermining the surgical advantage and potentially exacerbating neurological deficits, with a risk of mortality (4). Consequently, preventing the expansion of postoperative secondary hematomas is a significant problem to be addressed during stereotactic surgery. Point signals within the hematoma are frequently employed to accurately forecast the probability of primary hematoma expansion in patients with cerebral hemorrhage (5–7). Clinical studies regarding the prediction of secondary hematoma expansion following stereotactic surgery for basal ganglia hematoma remain unreported. This study aims to assess the predictive influence of CTA angiography point signals on secondary hematoma expansion following stereotactic surgery and to offer guidance for surgical treatment choices for moderate basal ganglia hematoma.

## 2 Data and methods

### 2.1 General information

A retrospective analysis was conducted on the clinical data of 143 patients with moderate basal ganglia hematoma who were admitted to the Department of Neurosurgery at Baise People’s Hospital from January 2021 to December 2022 and underwent stereotactic surgery combined with urokinase treatment within 6 to 48 h of disease onset. Criteria inclusion: ① Patients between 18 and 80 years of age with no prior history of cerebral hemorrhage. ② Patients diagnosed with basal ganglia hematoma, according to the 2022 American Heart Association/American Stroke Association guidelines (8), with no prior history of cerebral hemorrhage. ③ Hematoma volume 30–60 mL with or without rupture into the ventricle. ④ Head CT and CTA were performed 6 to 48 h post-onset, and the head computed tomography (CT) evaluation was conducted within 24 h following surgery. ⑤ In CTA pictures, the internal point sign of the hematoma is depicted as negative, indicating the absence of ongoing bleeding, thereby excluding individuals with primary hematoma expansion.

Exclusion criteria: ① Individuals with subsequent cerebral hemorrhage resulting from intracranial aneurysm, cerebrovascular malformation, stroke due to brain tumor, traumatic brain injury, etc. ② Patients exhibiting compromised coagulation function as a result of prolonged administration of anticoagulants, including aspirin and clopidogrel. ③ Concomitant failure of the heart, liver, kidneys, and other organs, associated with a reduced prognosis. Patients satisfying the aforementioned criteria and receiving stereotactic surgery guided by CTA angiography point sign constitute the experimental group, whereas those undergoing stereotactic surgery guided by conventional CT imaging form the control group. Sample size justification: in the pilot experiment, the occurrence of secondary hematoma expansion events was 0.01 in the experimental group and 0.19 in the control group. The PASS 15.0 software calculation indicates that each cohort of 54 patients can achieve 90% effectiveness with an alpha level of 0.025. The present investigation comprises 143 patients, which is adequate to guarantee sufficient statistical power to identify clinically meaningful intergroup differences. The Ethics Committee of Baise People’s Hospital approved the study protocol. The patients or their guardians signed the informed consent for treatment. The patient selection flow chart is as follows:

**Table:** 

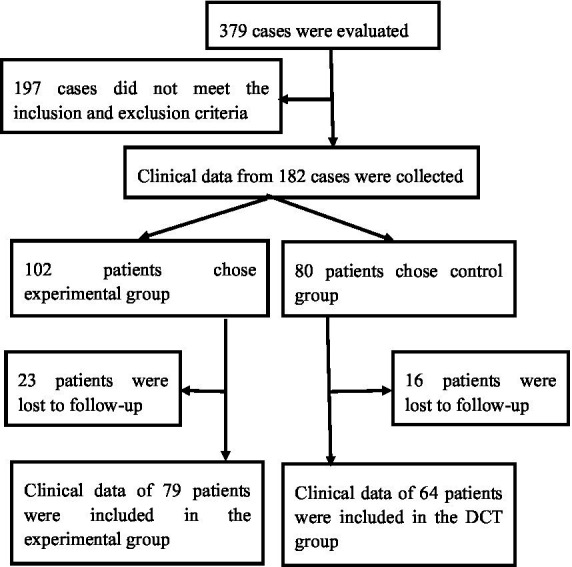

### 2.2 Observation and assessment criteria

**Figure 1:**
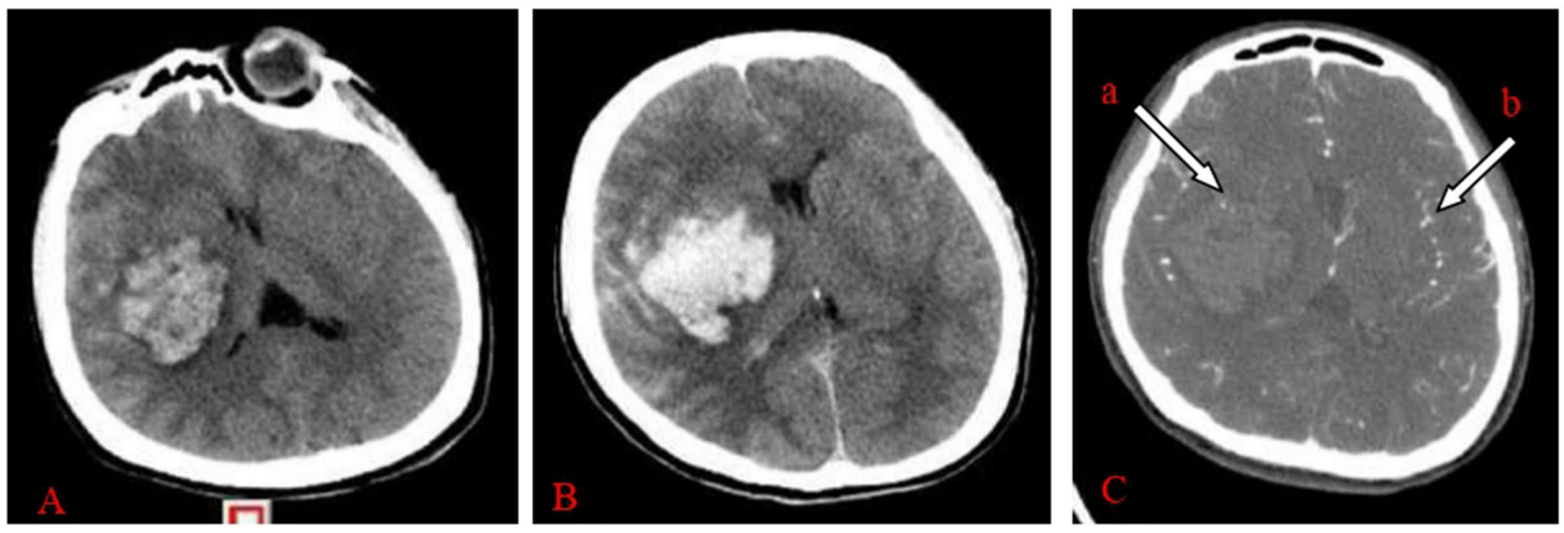
**(A–C)** Female, 47 years old, experienced a sudden headache accompanied by left leg paralysis for 4 h and hemorrhage in the right basal ganglia region. **(A)** Initial CT imaging of the right basal ganglia region revealed an anomalous hyperdense shadow, next to the internal capsule, indicating compression of the internal capsule. The Remebot software system immediately calculated the hematoma volume to be approximately 50.17 mL. **(B)** CTA reveals a hematoma including an internal hematoma, with (a) arrowheads denoting punctate hyperdense enhancement, confirming a positive “hematoma internal point sign.” (b) Arrowheads denote point-like and striated hyperintensity increase, signifying a positive “angiographic point sign.” Upon reviewing the CT plain scan after 6 h of onset, the hemorrhage in the right basal ganglia region had risen, with the Remebot software system automatically measuring the hematoma volume at approximately 57.92 mL.

Clinical outcomes were systematically assessed at two different time points. Short-term outcomes, including Glasgow Coma Scale (GCS) score, postoperative complications and mortality, were assessed during the 30-day postoperative follow-up period. The Modified Rankin Scale (MRS) score was analysed for long-term outcomes during the 6-month postoperative follow-up period. Assessment of hematoma volume: raw cranial CT (Figure 1A) or CTA images were uploaded into the Remebot software system, where the hematoma boundaries were manually delineated, and the software automatically computed the hematoma volume. The criteria for primary hematoma expansion (Figure 1C) are: persistent bleeding within the hematoma, an increase in hematoma volume above 33% relative to the prior volume, or an increase greater than 6 mL compared to the hematoma volume identified during the initial CT scan. The secondary hematoma expansion parameters are as follows: ① No hematoma formed along the trajectory of the preoperative drain puncture. ② An increase in hematoma volume exceeding 33% of the preoperative volume or more than 6 mL above the initial hematoma volume postoperatively.③ the presence of hematoma or subdural hematoma encircling the drainage tube. ④ The occurrence of new hemorrhage on postoperative imaging or notable clinical deterioration indicative of hematoma expansion. In cases of active hemorrhage, the initial head computed tomography angiography (CTA) reveals a speckled or irregular hyperdense appearance within the hematoma, indicative of contrast agent extravasation resulting from blood vessel rupture. This image exhibits limited dimensions, lacks continuity, and is not associated with external blood vessels, representing the internal point sign of the hematoma (Figure 1B-a) (5, 6). The intravascular contrast agent exhibited speckled or striated hyperdense pictures on the original CTA images, referred to as the angiographic point sign (Figure 1B-b). The “angiographic spot sign” is distinct from the “internal point sign” referenced in the literature, which often denotes hyperintense signals within the hematoma and is frequently utilized as a marker for current bleeding. In our study, the “angiographic spot sign” denotes hyperintense signals seen outside the hematoma, which informed the surgical strategy in the experimental group. According to the Modified Rankin Scale (MRS), grades 1–4 are deemed effectual, whereas grade 5 is considered ineffectual (9). All clinical data were independently documented by two seasoned neurosurgeons, with a third senior neurosurgeon serving as an arbitrator in the event of a dispute.

### 2.3 Surgical methods

**Figure 2:**
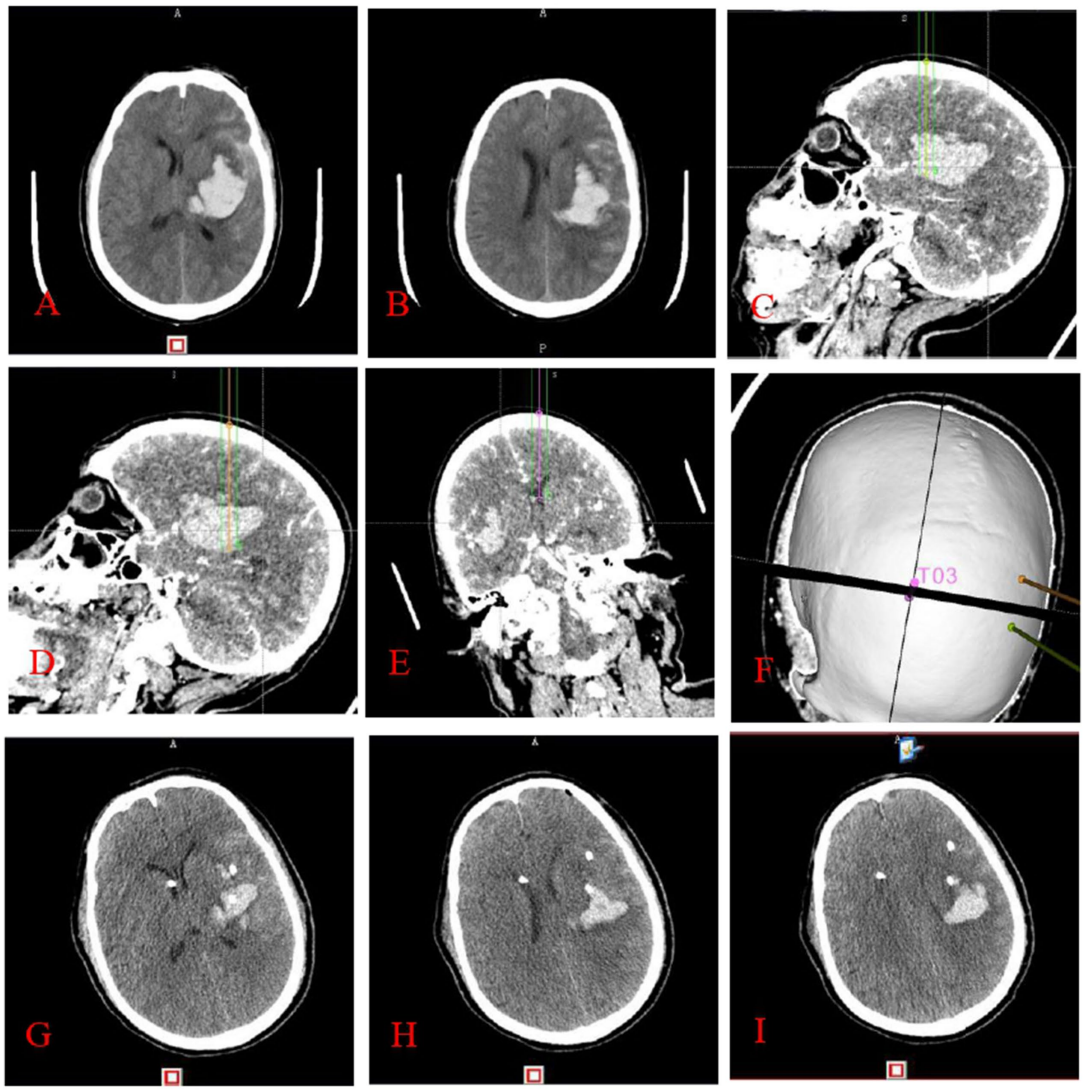
This figure illustrates stereotactic surgery conducted by the principle of cranial CTA angiography point sign: preoperative cranial CT **(A,B)** indicates a left basal ganglia hematoma. The surgical route design **(C–F)** utilized the anterior midpoint **(C)**, posterior midpoint **(D)**, and anterior horn of the right ventricle **(E)** of the hematoma as target areas, with puncture placement and drainage conducted with the aid of a neurosurgical robot. It is noteworthy that the dotted or striated hyperdense shadows depicted in images **C–E** correspond to angiographic points on the CTA, which are instrumental in the design of the surgical pathway. Three-dimensional reconstruction image of the puncture trajectory **(F)**. Cranial CT **(G–I)** conducted within 24 h post-surgery indicates a substantial decrease in the hematoma.

**Figure 3:**
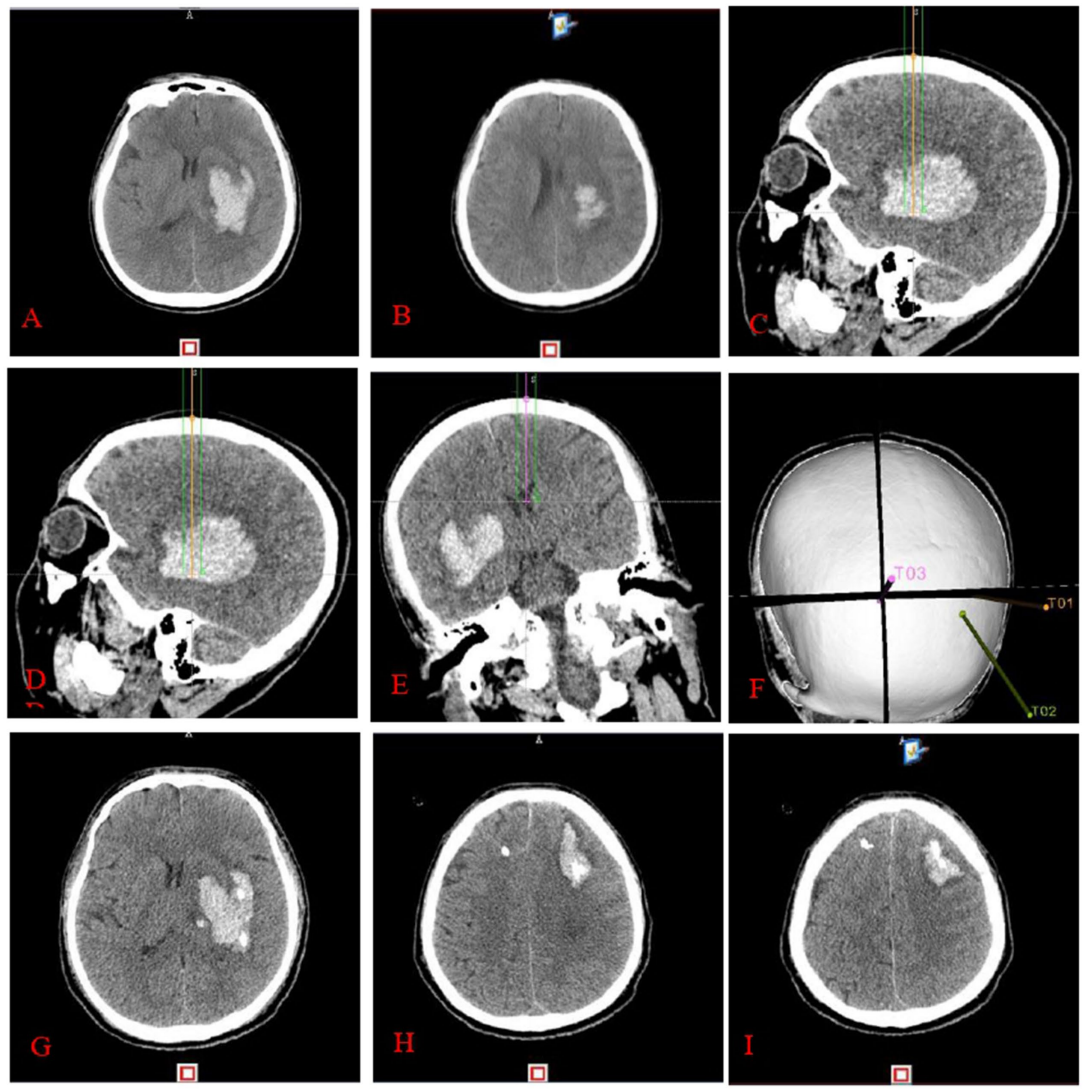
Completion of stereotactic surgery guided by cranial CT: preoperative cranial CT **(A,B)** reveals a left basal ganglia hematoma. The surgical route design **(C–F)** involved targeting the anterior midpoint **(C)**, posterior midpoint **(D)**, and anterior pedicle of the right ventricle **(E)** of the hematoma, with puncture placement and drainage performed by a neurosurgical robot. Three-dimensional reconstruction image of the puncture pathway **(F)**. Cranial CT **(G–I)** performed within 24 h postoperatively reveals an enlarged hematoma and hematoma formation around the drainage tube.

The experimental group underwent a thin-layer CTA scan of the head after applying ceramic head marking points before surgery. The original pictures were then transferred into the neurosurgical robot Remebot software system. Angiographic point markers is used to analyse the contrast build-up on the CTA image on the side of the hematoma and identify potential vessels that may be at risk during the procedure. Consequently, precise surgical path planning can be executed, intentionally circumventing these vessels and mitigating the danger of secondary hematoma expansion (Figure 2). Conversely, the control group employed the conventional technique for surgical path planning: following the application of ceramic markers preoperatively, a thin-layer CT scan was conducted, and subsequently, the raw CT images were integrated into the Remebot neurosurgical robotic software system, culminating in the design of the surgical path without the utilization of angiographic spot markers (Figure 3).

In both cohorts, the hematoma’s center served as the midpoint, while the vertical midline established a plane to bifurcate the hematoma into anterior and posterior sections. Consequently, the midpoints of these two segments were identified as the target points for multipath puncture and drainage procedures along the longitudinal axis of the hematoma using the frontal and temporal approaches to enhance hematoma drainage. Upon the conclusion of general anesthesia, the head was stabilized with a Medfield head frame, succeeded by patient registration, robotic arm registration, and optical registration in that sequence.

Following concluding the sterilization in accordance with established protocols, the skin puncture sites in the frontal and temporal areas are identified based on the preoperative surgical pathway established by the Remebot software system. An incision approximately 1.0 cm in length was created at the puncture point’s center, followed by the formation of a bone hole around 0.5 centimeters in diameter using a cranial drill. Two 12F soft silicone tubes were inserted into the centers of the anterior and posterior hematomas under robotic arm guidance. The drainage tubes were then extended through two subcutaneous tunnels measuring 5.0 cm long and secured after approximately 60% of the hematoma was aspirated slowly with a 5 mL syringe. Extraventricular drainage was conducted in both groups following the puncture and draining of the hematoma to prevent the development of obstructive hydrocephalus. After successfully inserting the tube, the skin incision was sutured in layers. A cranial CT was conducted within 24 h after post-surgery, and 20,000 units of urokinase were administered into the center of the anterior and posterior hematoma via two drainage tubes. The drainage tubes were subsequently opened after being occluded for 2–3 h. The cranial CT was assessed every 2–3 days, and the drainage tubes were extracted when the residual hematoma volume fell below 10% of the preoperative hematoma volume.

### 2.4 Statistical processing

The statistical analysis was conducted using SPSS version 20.0 software. Data were represented as percentages, and the *χ*^2^ test was employed for one-way analysis. The measurement data were verified for normal distribution and presented as $\bar{x\;}\pm s$ and the *t*-test of two independent samples was used. A *p*-value less than 0.05 indicated that the difference was statistically significant.

## 3 Results

### 3.1 Analysis of diverse parameters influencing postoperative secondary hematoma expansion in patients

Among 143 patients with moderate basal ganglia hematoma who underwent stereotactic surgery, a total of 12 patients had an event of postoperative secondary hematoma expansion. Among these, five cases (6.58%) were male and seven cases (10.45%) were female. Eight cases (10.67%) involved patients aged ≥60 years, while four cases (5.88%) involved patients aged <60 years. Six cases (8.45%) had a Glasgow Coma Scale (GCS) score of ≤8 at admission, and six cases (8.33%) had a GCS score of >8. Four cases (6.70%) presented with subarachnoid hemorrhage, whereas eight cases (9.41%) did not. Ventricular rupture occurred in five cases (8.20%), while seven cases (8.54%) did not exhibit ventricular rupture. Midline displacement was observed in eight cases (11.59%), and four cases (5.41%) showed no displacement. Hematoma configuration varied, with three cases (5.45%) exhibiting a non-uniform configuration, six cases (10.17%) showing a circular configuration, and three cases (10.34%) categorized as other configurations. A one-way analysis revealed no statistically significant differences (*p* > 0.05). None of the patients who underwent CTA-guided stereotactic surgery experienced postoperative secondary hematoma expansion, while all 12 patients who did experience such expansion had undergone CT-guided stereotactic surgery; one-way analysis revealed a statistically significant difference (*p* < 0.05). Refer to Table 1.

**Table 1:** Comparison of various causal factors affecting the rate of postoperative secondary hematoma growth in patients (*n*, %).

Contributing factors		Cases (*n*)	Postoperative secondary hematoma expansion (*n*, %)	*χ*^2^ value	*p*-value
Gender	Male	76	5 (6.58)	0.693	0.548
	Female	67	7 (10.45)		
Age (years)	≥60	75	8 (10.67)	1.062	0.374
	<60	68	4 (5.88)		
GCS score at admission (score)	≤8	71	6 (8.45)	0.001	1.000
	>8	72	6 (8.33)		
Subarachnoid hemorrhage	Yes	58	4 (6.70)	0.284	0.762
	No	85	8 (9.41)		
Ventricular rupture	Yes	61	5 (8.20)	0.005	1.000
	No	82	7 (8.54)		
Displacement of the midline	Yes	69	8 (11.59)	1.779	0.233
	No	74	4 (5.41)		
Configuration of hematoma	Non-uniform	55	3 (5.45)	1.004	0.605
	Circular	59	6 (10.17)		
	Others	29	3 (10.34)		
Intraoperative navigation	CTA	79	0	16.169	<0.001
	CT	64	12 (18.75)		

### 3.2 The short-term clinical efficacy at 30 days and the long-term clinical efficacy at 6 months were compared between the two groups

The experimental group experienced 27 postoperative lung infections (34.18%), whereas the control group had 33 infections (51.56%). The average GCS score was (9.46 ± 2.23) in the experimental group and (7.94 ± 4.68) in the control group. The mortality rate was 2 (2.53%) in the experimental group and 8 (12.50%) in the control group. The treatment efficacy rate (MRS) at 6 months was 59 cases (74.68%) in the experimental group and 35 cases (54.69%) in the control group. The disparity between the two groups was statistically significant (*p* < 0.05). The occurrence of postoperative cerebral infection in the experimental and control groups was two cases (2.53%) and one case (1.56%), respectively, with no statistically significant difference observed between the two groups (*p* > 0.05). Refer to Table 2.

**Table 2:** Comparison of postoperative clinical outcomes across the two patient groups.

Clinical efficacy		Experimental group (*n* = 79)	Control group (*n* = 64)	*t*/*χ*^2^ value	*p*-value
GCS score ( $\bar{x\;}\pm s$ , score)		9.46 ± 2.23	7.94 ± 4.68	2.553	0.012
Mortality (*n*, %)		2 (2.53)	8 (12.50)	4.935	0.044
Postoperative complications (*n*, %)	Lung infections	27 (34.18)	33 (51.56)	4.388	0.042
	Intracranial infections	2 (2.53)	1 (4.56)	0.162	1.000
Efficacy rate (MRS, score)		59 (74.68)	35 (54.69)	6.276	0.014

## 4 Discussion

Patients with basal ganglia hematomas typically experience permanent paralysis attributed to the impairment of brain function resulting from the hematoma, with hematoma expansion being a significant determinant of the patient’s prognosis (4). Approximately 20% of patients experienced hematoma expansion events in the initial phase (<6 h of beginning), leading to significant deterioration of their status and unfavorable prognosis (10). 70% of hematoma expansion incidents transpired within 3 h of beginning, with the majority resulting in poor prognoses or mortality (11). Consequently, the prompt removal of the hematoma and the prevention of its expansion has emerged as critical strategies to mitigate mortality and disability rates. Patients exhibiting a hematoma volume of 30–60 mL demonstrate a negligible response to pharmacological treatment alone and generally necessitate surgical intervention alongside combination therapy. The primary surgical techniques now employed in clinical practice are craniotomy and minimally invasive surgery. The benefits of rapid hematoma removal through surgery are largely offset by the brain tissue damage and associated postoperative complications caused by craniotomy. Consequently, minimally invasive surgery has emerged as one of the most prevalent surgical techniques for the management of cerebral hemorrhage and has been widely adopted and implemented. Currently, robot-assisted stereotactic surgery in neurosurgery is extensively utilized across various domains, particularly in the management of moderate basal ganglia hematoma. In the surgical management of patients with moderate-volume basal ganglia hematoma, the surgeon employs a robotic surgical software system to strategize the puncture trajectory before the procedure, subsequently utilizing real-time navigation to locate the center of the hematoma during surgery accurately. A multipath puncture and drainage technique was employed to enable hematoma drainage. Despite significant advancements in the precision of stereotactic surgery facilitated by neurosurgical robots, postoperative secondary hematoma expansion continues to occur sometimes. Consequently, predicting subsequent hematoma expansion following stereotactic surgery and utilizing this predictive tool to inform surgical strategy has emerged as a prominent research focus for neurosurgeons.

The internal hematoma point sign significantly predicts primary hematoma expansion and is incorporated in the 2015 American Heart Association/American Stroke Association guidelines for cerebral hemorrhage (12). The contrast density of the point sign exceeds 1.5 mm, which is more than double the density of the background hematoma. The initial cranial CT scan does not exhibit a hyperdense shadow at the appropriate area. A comprehensive retrospective study revealed that 81 of 268 patients with cerebral hemorrhage tested positive for the dot sign, exhibiting an average hematoma volume increase of 8.6 mL. In contrast, the remaining 187 patients, who were negative for the dot sign, demonstrated an average hematoma volume increase of 0.4 mL (13). Prior research reveals that a positive internal point sign in a hematoma signifies that the vessel responsible for the hemorrhage has not been fully occluded, leading to ongoing active bleeding (14, 17). A positive internal point signal in hematomas as an important method for forecasting hematoma expansion in patients within 6 h of disease onset (15, 18). The point sign demonstrates an 85.7% accuracy in forecasting hematoma expansion when the hematoma volume surpasses 30 mL (16). Moderate-volume basal ganglia hematomas typically necessitate surgical intervention, and the internal point sign of the hematoma serves as a criterion for determining the surgical strategy. Despite its significant surgical risks and postoperative problems, craniotomy can achieve efficient hemostasis of active hemorrhages through direct visualization. Consequently, the authors determined that patients exhibiting a positive internal point sign of hematoma should undergo craniotomy to mitigate the risk of postoperative hematoma extension. Exhibiting negative internal point signatures of hematomas signifies the cessation of the bleeding process within the hematoma, indicating that postoperative secondary hematoma extension can be entirely prevented in the absence of vascular injury during surgery. In patients exhibiting negative internal point signatures of hematomas, stereotactic surgery, guided by angiographic point signatures, is believed to effectively avoid postoperative secondary hematoma expansion. Furthermore, the accuracy of the surgical procedure, the postoperative management of blood pressure, and the judicious application of hemostatic agents continue to have a significant and beneficial role in preventing the expansion of secondary hematomas.

This study exclusively selected individuals exhibiting a negative internal point sign of the hematoma 6 h from onset, indicating the absence of primary hematoma expansion that could have influenced the study’s outcomes. Consequently, all patients exhibiting increased hematomas experienced subsequent expansion from intraoperative vascular damage. The experimental group notably circumvented blood vessels by adeptly employing CTA angiographic point labeling in surgical path planning with the Remebot software system. This technique demonstrated significant efficacy in averting intraoperative vascular damage and hematoma expansion. In this study, the experimental group exhibited no secondary hematoma expansion, while 12 patients (18.75%) in the control group experienced secondary hematoma expansion post-surgery. The angiographic point sign may predict the probability of secondary hematoma expansion during stereotactic surgery. This study employed multipath puncture placement to enable drainage for hematoma resolution, enhancing clinical outcomes; nevertheless, this approach concurrently elevated the likelihood of subsequent hematoma expansion post-surgery in the control group. Shen et al. demonstrated that hematoma expansion following minimally invasive surgery is significantly associated with patient prognosis (16, 19). This study’s innovation lies in predicting the risk of secondary hematoma expansion following stereotactic surgery for basal ganglia hematomas by employing cranial CTA angiographic point signatures, thereby enhancing patient clinical outcomes through high-precision stereotactic surgical strategies. In this study, the control group exhibited a GCS score of 7.94 ± 4.68 on day 30, with a mortality rate of eight cases (12.50%) within 30 days. Conversely, the experimental group demonstrated a GCS score of 9.46 ± 2.23 on day 30 and a mortality rate of two cases (2.53%) within the same timeframe. A statistically significant difference was seen between the two groups (*p* < 0.05), indicating that the short-term clinical efficacy of the experimental group was much superior to that of the control group. The incidence of postoperative lung infection and cerebral infection in the experimental group was 27 cases (34.18%) and two cases (2.53%), respectively, while in the control group, it was 33 cases (51.56%) and one case (1.56%). A statistically significant difference was observed in comparing postoperative pulmonary infections between the two groups (*p* < 0.05). This signifies that the probability of postoperative lung infection was markedly greater in the control group compared to the experimental group. However, no statistically significant difference was found when postoperative cerebral infections were compared between the two groups (*p* > 0.05). This signifies that there is no substantial disparity in the occurrence of postoperative cerebral infections between the two groups. The long-term efficacy was assessed 6 months after disease onset in individuals with basal ganglia hematomas utilizing the efficacy rate of MRS score. This study revealed that the effective rates for the experimental and control groups were 59 cases (74.68%) and 35 cases (54.69%), respectively. A statistically significant difference was observed between the two groups (*p* < 0.05), indicating that the long-term clinical efficacy of the experimental group was markedly superior to that of the control group.

## 5 Study limitations

This research is a retrospective clinical case study. The authors meticulously adhered to the inclusion and exclusion criteria for case selection and thoroughly gathered clinical data from both patient groups to mitigate the influence of selection bias on the study outcomes. As a retrospective single-center analysis with a limited sample size, this study has inherent limitations that necessitate prompt validation through future prospective multicenter investigations with larger cohorts. To address these limitations and improve the validation of the findings, additional multicenter, large-sample prospective studies are essential to minimize selection bias and enhance the generalizability of the results.

## 6 Conclusion

In summary, the cranial CTA angiographic point sign is a reliable predictor of subsequent hematoma expansion following stereotactic surgery and plays a significant role in the stereotactic surgical management of moderate-volume basal ganglia hematomas. The cranial CTA angiographic point sign should be prioritized in surgical treatment to develop a tailored surgical strategy, reducing the risk of postoperative secondary hematoma expansion while enhancing clinical prognosis and postoperative survival quality for patients.

## Data Availability

The original contributions presented in the study are included in the article/supplementary material, further inquiries can be directed to the corresponding author.
